# Efficacy and safety of transcatheter left atrial appendage closure guided by transoesophageal echocardiography using standard and micro TEE probes: a single-center retrospective study

**DOI:** 10.3389/fcvm.2026.1855910

**Published:** 2026-06-09

**Authors:** Alessandro Barbarossa, Francesca Coraducci, Elisa Nicolini, Laura Cipolletta, Alessandro Maolo, Michela Casella, Fabio Vagnarelli, Federico Guerra, Marco Marini, Antonio Dello Russo, Tommaso Piva

**Affiliations:** 1 Cardiology and Arrhythmology Clinic, Cardiovascular Department, Azienda Ospedaliero Universitaria (AOU) Delle Marche, Ancona, Italy; 2 Cardiology Division-Intensive Cardiac Care Unit, Cardiovascular Department, Azienda Ospedaliero Universitaria (AOU) Delle Marche, Ancona, Italy; 3 Interventional Cardiology Unit, Division of Cardiology, Azienda Ospedaliero Universitaria (AOU) Delle Marche, Ancona, Italy.; 4 Department of Clinical Sciences, Marche Polytechnic University, Ancona, Italy; 5 Maria Cecilia Hospital, GVM Care & Research, Cotignola, Italy; 6 Department of Biomedical Sciences and Public Health, Marche Polytechnic University, Ancona, Italy

**Keywords:** atrial fibrillation, echocardiography, left atrial appendage closure, micro transoesophageal echocardiography probe, transoesophageal echocardiography

## Abstract

**Background:**

Transcatheter left atrial appendage closure (T-LAAC) represents an effective alternative to long-term anticoagulation in patients with atrial fibrillation (AF) and contraindications to oral anticoagulants. Transoesophageal echocardiography (TEE) is the standard imaging modality for procedural guidance, while micro-TEE may allow adequate imaging under conscious sedation, potentially simplifying procedural workflow. This study aimed to evaluate the efficacy and safety of micro-TEE compared with standard TEE guidance during T-LAAC.

**Methods:**

Single-center, retrospective study, all consecutive patients undergoing T-LAAC between June 2021 and August 2024. Patients were stratified according to intraprocedural imaging guidance (micro-TEE vs. standard TEE). Baseline characteristics, procedural data, and short-term outcomes were analyzed. A subgroup analysis excluding patients who underwent simultaneous combined atrial fibrillation ablation procedures was performed to reduce confounding.

**Results:**

A total of 47 patients were considered; one was excluded due to poor view in both imaging modalities, for a total of 46 patients. 38 procedures were performed under standard TEE and 8 under micro-TEE guidance. No differences in baseline clinical and echocardiographic characteristics were found. Procedural success was similar, with one failure in the standard TEE group due to the unavailability of the right size of the closure device. No significant differences were observed in periprocedural complications, including mild pericardial effusion, cardiac tamponade, device-related thrombosis, or significant peridevice leak. Micro-TEE guidance was associated with a significantly shorter procedural time (153 vs. 175 min, *p* = 0.040) and a lower rate of orotracheal intubation, without increases in fluoroscopy time, radiation exposure, or contrast use. This data was confirmed even after considering only isolated T-LAAC procedures, excluding simultaneous combined AF ablation-T-LAAC procedures (143 vs. 160 min, *p* = 0.042). At 1-month follow-up, the micro-TEE group did not show an increased complication rate.

**Conclusion:**

The use of micro-TEE during T-LAAC procedures did not show a significant difference in procedural success rate and was not associated with an increased complication rate compared with standard TEE guidance. The use of micro-TEE showed a reduction in total procedural time, partly due to the reduced need for OTI, without an increase in radiation exposure, fluoroscopy time, and contrast use.

## 1 Introduction

Atrial fibrillation (AF) is the most common cardiac arrhythmia worldwide and is associated with a significantly increased risk of stroke and systemic thromboembolism (1). Oral anticoagulation remains the cornerstone of stroke prevention; however, a substantial proportion of patients have contraindications, experience bleeding complications, or demonstrate poor long-term adherence (2, 3). In patients with non-valvular AF, more than 90% of thrombi originate from the left atrial appendage (LAA), providing the rationale for left atrial appendage closure (LAAC) as an alternative strategy for stroke prevention (4). Transcatheter LAAC (T-LAAC) has emerged as a safe and effective alternative to long-term anticoagulation in selected patients, supported by randomized controlled trials (RCTs) demonstrating comparable stroke prevention with reduced long-term bleeding risk (5–9). Current European guidelines suggest that T-LAAC may be considered in patients with AF and contraindications to long-term anticoagulation (1).

Intraprocedural imaging is essential to guide transseptal puncture, ensure coaxial alignment between the delivery system and the LAA, and confirm device positioning while excluding complications such as device leaks or device-related thrombosis (DRT). Transoesophageal echocardiography (TEE) and fluoroscopy are the standard imaging modalities for procedural guidance. However, newer alternatives are emerging, such as intracardiac echography (ICE), which allows the complete avoidance of general anesthesia and facilitates faster patient discharge (10). However, ICE adoption remains limited due to a longer learning curve, cost, and limited validation of release criteria and limited procedural views.

On the other hand, TEE use is generally safe and low-cost but typically requires general anesthesia with orotracheal intubation (OTI) to improve patient tolerance to the probe, potentially resulting in increased costs, prolonged procedural and recovery times, and higher risk of anesthesia-related complications.

Recently, smaller transoesophageal probes (mini-TEE and micro-TEE) have been introduced and may carry the benefit of TEE and ICE together: these probes allow imaging monitoring under conscious or mild sedation, potentially avoiding general anesthesia and OTI, while maintaining adequate image quality. Unlike ICE, micro-TEE and mini-TEE probes are low cost, reusable, and do not require additional venous access.

The aim of our study was to evaluate the efficacy and safety of percutaneous left atrial appendage closure performed under micro-TEE guidance compared with the procedure performed under standard TEE guidance.

## 2 Materials and methods

In this single-center retrospective study conducted at the Azienda Ospedaliero-Universitaria delle Marche, we enrolled all consecutive patients who underwent T-LAAC, either as a standalone procedure or in combination with atrial fibrillation ablation with pulmonary veins isolation, between June 2021 and August 2024.

Baseline characteristics, including demographic data and pre-procedural echocardiographic parameters, as well as procedural data and short-term follow-up outcomes, were collected. At one-month follow-up, TEE was used to assess for any complications.

Patients were stratified into two groups according to the type of echocardiographic guidance used during the procedure: a micro-TEE probe (model S8-3T, Koninklijke Philips N.V., Amsterdam, the Netherlands) versus a standard TEE probe (model X8-2T, Koninklijke Philips N.V., Amsterdam, the Netherlands). The main differences between the two probes are summarized in Table 1.

**Table 1:** Technical characteristics of standard and micro-TEE probes (Koninklijke Philips N.V., Amsterdam, The Netherlands).

Probe characteristics	Standard TEE (X8-2t)	Micro TEE (S8-3t)
Shaft diameter	9.5 mm	5.2 mm
Tip diameter	16.6 mm	7.5 mm
Elements	> 2,500	32
Frequency range	7–2 MHz	8–3 MHz
3D acquisition	yes	no
Shaft length	106 cm	80 cm

TEE, transoesophageal echocardiography.

The choice between micro-TEE and standard TEE, as well as the choice of OTI, was left to the clinical discretion of the echocardiographer and anesthesiologist, also considering the feasibility of conscious sedation vs. general anesthesia, without predefined patient selection criteria; therefore, potential selection bias cannot be excluded.

In the case of micro-TEE, the probe was placed after obtaining vascular access but before transeptal puncture; in the case of standard TEE, the probe was placed immediately after OTI, before obtaining vascular access.

Procedural outcomes were compared between the groups in terms of efficacy, safety, and procedural characteristics, including fluoroscopy time and procedure duration. Procedure duration was calculated from entry into the Cath-lab to the end of the procedure, including the time required to secure vascular access. Procedural success was defined as successful device implantation in the LAA with adequate positioning and sealing (peri-device leak ≤ 5 mm), without major procedure-related complications, including device embolization, pericardial effusion requiring intervention, mild pericardial effusion (< 1 cm not requiring pericardiocentesis and without hemodynamic instability), stroke, major bleeding, or in-hospital death. A subgroup analysis excluding patients who underwent combined atrial fibrillation ablation and T-LAAC was also performed to minimize the potential confounding effect of ablation on procedural times.

## 3 Statistical analysis

Continuous variables were expressed as mean ± standard deviation when a normal distribution could be assumed, and as median with interquartile range for variables not normally distributed. Comparisons between groups for continuous variables were performed using the Student's t-test when normal distribution was assumed or the Mann–Whitney U test when normality could not be assumed. Normality was assessed using the Kolmogorov–Smirnov test or the Shapiro–Wilk test, as appropriate. Categorical variables are presented as frequencies and percentages and were compared using the chi-square test or Fisher's exact test, as appropriate. An alpha level of *p* < 0.05 (two-tailed) was considered significant.

## 4 Results

A total of 47 patients who underwent T-LAAC between June 2021 and August 2024 were identified and included in the analysis. One patient required crossover from micro-TEE to standard TEE at the beginning of the procedure due to suboptimal visualization; however, the procedure was subsequently aborted because adequate imaging could not be obtained with standard TEE as well, and the patient was therefore excluded from the study. In this patient, pre-procedural evaluation was performed with cardiac computed tomography alone. The final number of patients enrolled is 46, of which 38 procedures were performed under standard TEE guidance and 8 under micro-TEE guidance.

The two populations did not show significant differences in baseline demographic characteristics (Table 2). The mean age was 76 ± 8 years, with a female prevalence of 30%. No differences were observed between the two groups regarding atrial fibrillation type or indication for T-LAAC. Patients had a high thromboembolic risk, with a median CHA₂DS₂-VA score of 4 in both groups (*p* = 0.604), and an increased bleeding risk, with a median HAS-BLED score of 2 in the micro-TEE group and 3 in the standard TEE (*p* = 0.184). No significant differences were found with respect to key baseline echocardiographic characteristics, including left ventricular ejection fraction (median LVEF 55%, IQR 45–62), left atrial end systolic volume indexed (median LAESVi 49 mL, IQR 37–62), and left atrial appendage morphology (Table 3).

**Table 2:** Baseline characteristics of the study population according to the type of TEE probe used.

Baseline characteristics	Overall (*n* = 46)	Micro TEE (*n* = 8)	Standard TEE (*n* = 38)	*p*-value
Age (years)	76 ± 8	75 ± 8	76 ± 8	0.876
Height (cm)	1.68 ± 0.07	1.73 ± 0.05	1.69 ± 0.08	0.156
Weight (kg)	77 ± 14	76 ± 13	77 ± 14	0.956
BMI (kg/m2)	26 (23–29)	25 (23–28)	26 (24–30)	0.032
Female sex, *n*(%)	14 (30)	2 (25)	12 (32)	0.745
Creatinine (mg/dL)	1.00 (0.82–1.47)	1.40 (1.2–1.57)	1 (0.80–1.57)	0.126
eGFR (mL/min/1.73 m2)	57 (42–85)	46 (32–62)	63 (42–85)	0.229
Indication for closure, *n*(%)				0.416
Gastrointestinal bleeding	18 (38)	3 (38)	15 (38)	
Intracranial bleeding	12 (26)	3 (38)	9 (23)	
Cerebral amyloidosis/AV malformations/brain masses	4 (9)	0 (0)	4 (11)	
Recurrent stroke/persistent atrial thrombus	6 (13)	2 (25)	4 (10)	
Other	6 (13)	0 (0)	6 (15)	
Type of AF, n(%)				0.197
Paroxysmal	21 (46)	6 (75)	15 (39)	
Persistent	8 (17)	1 (13)	7 (18)	
Permanent	17 (36)	1 (13)	16 (41)	
CHA_2_DS_2_-VA	4 (3–5)	4 (3–4)	4 (3–5)	0.604
HAS-BLED	3 (2–3)	2 (2–2)	3 (2–3)	0.184
Combined AF ablation procedure, *n*(%)	9 (20)	2 (25)	7 (18)	0.644

TEE, transoesophageal echocardiography; AF, atrial fibrillation; BMI, body mass index; eGFR, estimated glomerular filtration rate; CHA₂DS₂-VA, stroke risk score in atrial fibrillation; HAS-BLED, bleeding risk score.

**Table 3:** Preprocedural echocardiographic characteristics of the study population according to the type of TEE probe used.

Preprocedural echocardiogram	Overall (*n* = 46)	Micro TEE (*n* = 8)	Standard TEE (*n* = 38)	*p*-value
Auricle morphology				0.623
Windsock	17 (36)	5 (62)	12 (31)	
Cauliflower	7 (15)	1 (13)	6 (15)	
Cactus	6 (13)	1 (13)	5 (13)	
Chicken-wing	10 (21)	1 (13)	9 (23)	
Unclassified/Other	6 (12)	0 (0)	6 (15)	
Landing zone (mm)	20 (17–21)	16 (16–21)	20 (17–21)	0.141
LAESVi (mL/m2)	49 (37–62)	42 (41–62)	50 (35–62)	0.862
LVEF (%)	55 (45–62)	57 (53–64)	55 (45–60)	0.448

LVEF, left ventricular ejection Fraction; LAVi, left atrial end systolic volume indexed.

Among the 46 patients undergoing T-LAAC, 37 underwent isolated procedures, whereas 9 underwent combined T-LAAC and atrial fibrillation catheter ablation with pulmonary veins isolation. The prevalence of patients undergoing isolated procedures vs. combined procedures did not differ between the two groups (2 patients in the micro-TEE group vs. 7 in the standard TEE group, *p* = 0.644). No differences were observed in the type of devices used (Table 4).

**Table 4:** Procedural acute complications and procedural data according to type of TEE probe used.

	Overall (*n* = 46)	Micro TEE (*n* = 8)	Standard TEE (*n* = 38)	*p*-value
Acute complications				
Procedural failure	1 (2)	0 (0)	1 (3)	0.415
Pericardial effusion	9 (19)	0 (0)	9 (23)	0.131
Cardiac tamponade	1 (2)	0 (0)	1 (5)	0.647
DRT	0 (0)	0 (0)	0 (0)	-
Leaks > 5 mm	2 (4)	0 (0)	2 (5)	0.513
Device type				0.668
Watchman FLX	29 (62)	6 (75)	23 (59)	
Amplatzer Amulet	14 (30)	2 (25)	12 (32)	
Omega	3 (6)	0 (0)	3 (8)	
Procedural data				
Total procedural time (min)	160 (150–200)	153 (131–160)	175 (150–200)	**0** **.** **040**
Total fluoroscopy time (min)	26 (19–33)	28 (14–29)	25 (19–36)	0.257
Total radiation dose (mGy·cm^2^)	127 (66–225)	174 (45–215)	121 (71–225)	0.267
Total contrast volume (mL)	96 (75–120)	100 (81–115)	91 (74–121)	0. 906
Oro-tracheal intubation	42 (93)	4 (50)	38 (100)	**<** **0.001**

DRT, device related thrombosis; TEE, transoesophageal echocardiography; mGy·cm^2^, milligray per square centimeter (unit of radiation dose).

Bold values indicate statistical significance (*p* < 0.05).

Procedural success was obtained in 45 patients. The reported procedural failure was due to inadequate LAA sizing, for which no appropriately sized device was available (Table 4).

**Figure 1:**
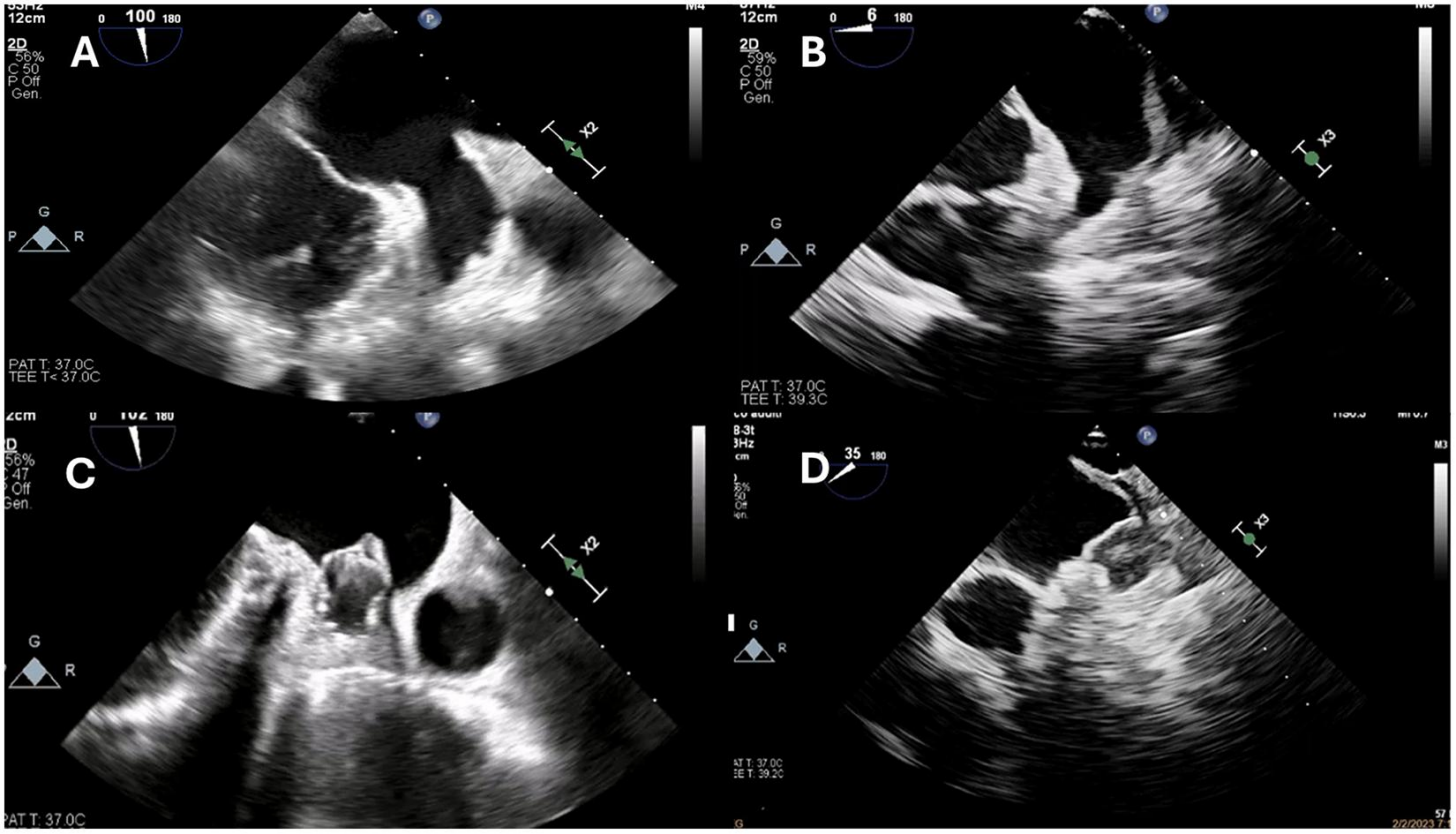
Comparison of imaging quality of standard TEE probe **(A,C)** and micro-TEE probe **(B,D)**. **(A)** LAA view with standard TEE probe. **(B)** LAA view with micro TEE probe. **(C)** View of closure device in LAA with standard TEE probe. **(D)** View of closure device in LAA with micro TEE probe. LAA, left atrial appendage; TEE, transoesophageal echocardiography.

In every patient in whom a micro-TEE probe was used, a high-quality view was obtained, and every procedure was successfully carried out. Figure 1 shows an example of echocardiographic images with standard TEE and micro-TEE.

Regarding acute procedural complications, no differences between micro-TEE and standard TEE groups were observed in terms of mild pericardial effusion (0 vs. 9, *p* = 0.131), cardiac tamponade (0 vs. 1, *p* = 0.647), DRT and device embolization (none in both groups), and peridevice leak > 5 mm (0 vs. 2, *p* = 0.513). The closure device used did not differ between the micro-TEE and standard TEE groups.

The OTI rate was significantly lower in patients undergoing micro-TEE-guided procedures (4 patients in the micro-TEE group vs. all patients in the standard TEE group; *p* < 0.001) (Table 4).

The use of micro-TEE did not increase fluoroscopy time (28 vs. 25 min, *p* = 0.257), radiation exposure (174 vs. 121 mGy·cm^2^, *p* = 0.267), or contrast medium use (100 vs. 91 mL, *p* = 0.906). Procedure duration was shorter in the micro-TEE group (153 min vs. 175 min *p* = 0.040). To avoid confounding factors associated with combined procedures, we performed a subgroup analysis excluding patients who underwent combined ablation (Table 5). This analysis demonstrated a significant reduction in total procedural time of approximately 20 min in the micro-TEE group (143 vs. 163 min, *p* = 0.041) without an increase in contrast use, radiation dose, or fluoroscopy times. (Table 5).

**Table 5:** Procedural times according to type of TEE probe used in patients who underwent isolated T-LAAC without atrial fibrillation ablation. .

Isolated T-LAAC	Overall (*n* = 37)	Micro TEE (*n* = 6)	Standard TEE (*n* = 31)	*p*-value
Procedural time (min)	160 (140–195)	143 (124–158)	160 (148–195)	**0** **.** **042**
Fluoroscopy time (min)	24 (16–33)	21 (13–28)	24 (19–35)	0.233
Total radiation dose (mGy·cm2)	118 (64–195)	115 (35–190)	118 (66–187)	0.634
Total contrast dose (mL)	96 (75–120)	100 (81–115)	91 (76–120)	0.869
Orotracheal Intubation	33 (89)	2 (33)	31 (100)	**<** **0.001**

T-LAAC, transcatheter left atrial appendage closure; mGy·cm^2^, milligray per square centimeter (unit of radiation dose).

Bold values indicate statistical significance (*p* < 0.05).

At one month, there were no differences in complication rates or ischemic stroke prevalence between groups. Moreover, all significant leaks > 5 mm resolved spontaneously within one month, and DRT occurred in two patients in the standard TEE probe group (Table 6).

**Table 6:** One-month follow-up outcomes of the study population according to type of TEE probe used.

1-Month Follow-up	Overall (*n* = 46)	Micro TEE (*n* = 8)	Standard TEE (*n* = 38)	*p*-value
Complications				
DRT	2 (4)	0 (0)	2 (5)	0.586
Leaks > 5 mm	0 (0)	0 (0)	0 (0)	-
Stroke/TIA	0 (0)	0 (0)	0 (0)	-

TEE, transoesophageal echocardiography; DRT, device-related thrombus; TIA, transient ischemic attack.

## 5 Discussion

Our study aimed to test the exploratory hypothesis that micro-TEE guidance was comparable to standard TEE guidance in terms of efficacy and safety in patients undergoing T-LAAC. However, several important limitations should be acknowledged. In particular, the imbalance between the two study groups reduces the statistical robustness of our findings. Moreover, because the choice of imaging modality was left to the clinical discretion of the echocardiographer and anesthesiologist rather than to predefined selection criteria, selection bias cannot be ruled out.

Despite potential selection bias and the aforementioned limitations, no significant differences were observed in baseline characteristics between the two groups. Likewise, no differences were reported in procedural success, intraprocedural complications, and 1-month clinical outcomes. Most notably, the use of micro-TEE did not increase fluoroscopy time, radiation exposure, or contrast use. Moreover, it was associated with lower procedural times, mainly driven by the reduced need for OTI, which was significantly lower in the micro-TEE group. This likely reduced the time from patient entry into the catheterization laboratory to the start of the procedure, as well as the recovery time after the procedure.

When patients undergoing combined T-LAAC and AF ablation were excluded from the analysis, since AF ablation duration depends on the energy source used (radiofrequency vs. electroporation), we found a significant reduction in procedural time of approximately 20 min and a lower rate of OTI with micro-TEE guidance, supporting previously reported findings.

At 1-month follow-up, no differences were observed in delayed complications. Notably, leaks > 5 mm detected immediately after the procedure resolved spontaneously within 1 month, whereas two cases of DRT were observed. It should be emphasized that our cohort represents a real-world population with both high ischemic and high hemorrhagic risk.

Other single-center studies have similarly demonstrated the feasibility and safety of these probes for guiding T-LAAC procedures without increasing procedural complications (11–13).

More recently, a multicentre observational study including 546 patients undergoing T-LAAC under conscious sedation with mini-TEE or micro-TEE guidance reported a technical success rate of 98%, with low rates of major periprocedural complications (2.9%) and minimal need for conversion to general anesthesia (0.7%). Short-term follow-up demonstrated low rates of significant residual leaks and device-related thrombosis (14). These data are in line with our findings suggesting that micro-TEE may represent a promising imaging strategy for T-LAAC, potentially simplifying procedural workflow while maintaining safety and efficacy.

## 6 Study limitations

This study has several limitations. First, due to the retrospective, observational, and non-randomized design, a selection bias cannot be excluded. Moreover, the difference in the two groups' numerosity must be acknowledged, and definitive conclusions must be interpreted with caution, with between-groups comparisons treated as exploratory analyses only. Another limitation is that both the choice of imaging modality and the use of OTI were left to the discretion of the echocardiographer and anesthesiologist, potentially introducing selection bias. Although all patients undergoing standard TEE-guided procedures required intubation, OTI was performed in the micro-TEE group according to the anesthesiologist's judgment. This heterogeneity may have reduced the observable impact of micro-TEE on procedural duration, particularly by limiting the potential time-saving effect related to avoidance of deep sedation and intubation.

## 7 Conclusions

The use of micro-TEE during T-LAAC procedures did not show a significant difference in procedural success rate and was not associated with an increased complication rate compared with standard TEE guidance. The use of micro-TEE showed a reduction in total procedural time, partly due to the reduced need for OTI, without an increase in radiation exposure, fluoroscopy time, and contrast use.

## Data Availability

The raw data supporting the conclusions of this article will be made available by the authors, without undue reservation.
